# Failure Rate of Single Dose Methotrexate in Managment of Ectopic Pregnancy

**DOI:** 10.1155/2015/902426

**Published:** 2015-03-12

**Authors:** Feras Sendy, Eman AlShehri, Amani AlAjmi, Elham Bamanie, Surekha Appani, Taghreed Shams

**Affiliations:** ^1^Obstetrics and Gynecology Department, King Fahad Medical City, Riyadh, Saudi Arabia; ^2^King Khalid University Hospital, King Saud University, Riyadh, Saudi Arabia; ^3^Obstetrics and Gynecology Department, King Abdulaziz Medical City, King Saud Bin Abdualziz University for Health Science (KSAU-HS), Riyadh, Saudi Arabia; ^4^Obstetrics and Gynecology Department, King Abdulaziz Medical City, King Saud Bin Abdulaziz University for Health Science (KSAU-HS), P.O. Box 9515, Jeddah 21423, Saudi Arabia

## Abstract

*Background*. One of the treatment modalities for ectopic pregnancy is methotrexate. The purpose of this study is to identify the failure rate of methotrexate in treating patients with ectopic pregnancy as well as the risk factors leading to treatment failure. *Methods*. A retrospective chart review of 225 patients who received methotrexate as a primary management option for ectopic pregnancy. Failure of single dose of methotrexate was defined as drop of BHCG level less than or equal to 14% in the seventh day after administration of methotrexate. *Results*. 225 patients had methotrexate. Most of the patients (151 (67%)) received methotrexate based on the following formula: f 50 mg X body surface area. Single dose of methotrexate was successful in 72% (162/225) of the patients. 28% (63/225) were labeled as failure of single dose of methotrexate because of suboptimal drop in BhCG. 63% (40/63) of failure received a second dose of methotrexate, and 37% (23/63) underwent surgical treatment. Among patient who received initial dose of methotrexate, 71% had moderate or severe pain, and 58% had ectopic mass size of more than 4 cm on ultrasound. *Conclusion*. Liberal use of medical treatment of ectopic pregnancy results in 71% success rate.

## 1. Introduction

Implantation of a fertilized ovum outside the uterine cavity is known as ectopic pregnancy (EP) [1–3]. It is a medical emergency due to the high morbidity and mortality in the reproductive age [4–7]. The incidence of ectopic pregnancies is 1-2% in the developed countries [8]. Ninety-eight percent of the implants are in the fallopian tube but also can be implanted at various sites such as ampulla, isthmus, ovaries, abdomen, and broad ligament [9, 10].

The etiology is uncertain but there are various risk factors, such as infertility, previous tubal surgery, contraceptive failure, cigarette smoking, and previous ectopic pregnancy [11]. Patients usually come with lower abdominal pain and vaginal bleeding from the 6th to 10th week of gestation [1]. It has been reported that one-third of patients have no clinical symptoms [12, 13]. If a patient came with syncope and signs of shock with a positive pregnancy test ruptured ectopic pregnancy should be suspected [11].

When the patient is pregnant, the physician should perform a work-up to detect possible ectopic or ruptured EP. Prompt ultrasound evaluation is the key in diagnosing ectopic pregnancy. Equivocal ultrasound results should be combined with quantitative beta subunit of human chorionic gonadotropin (BhCG) levels [14, 15]. If a patient has a BhCG level of 1,500 MIU per mL or greater, but the transvaginal ultrasonography does not show an intrauterine gestational sac, EP should be suspected [16].

While surgical approaches are the mainstay of treatment, advances in early diagnosis facilitated the introduction of medical therapy with methotrexate (MTX) in the 1980 [17–19]. Approximately 35% of women with EP are eligible for medical treatment [20]. The use of methotrexate (MTX) to treat early unruptured EP has been shown to be a safe and effective alternative to surgery in properly selected cases [21–23].

A Cochrane systemic review concluded that MTX treatment of EP had the highest success rate when plasma BhCG levels were below 3,000 IU/mL. It was stated that side effects from multiple-dose MTX treatment impaired quality of life [24]. In addition, MTX treatment was more expensive than laparoscopic salpingotomy when initial plasma-BhCG levels were above 3,000 IU/L [25]. Treatment with a single dose of MTX had fewer side effects, but the success rate was less than following a multiple-dose regimen [26].

Due to the routine use of early ultrasound among infertile patients who conceive, diagnosis of EP can be established early and medical treatment can be administered in most cases. The overall success rate of medical treatment in properly selected women is nearly 90% [27–29].

Our rational in this study was to review the cases that were diagnosed with EP and treated medically with MTX and observe the failure rate among them.

## 2. Methodology

Medical records of patients admitted to KAMC with a diagnosis of ectopic pregnancy which were retrospectively reviewed in the period between 2005 and 2011 were screened. Patients who had methotrexate as medical treatment of ectopic pregnancy were identified and included in this review. Patients who underwent surgical treatment as the first option of management were excluded.

The primary outcome measure of this review was the failure of first dose of methotrexate. Failure is defined in two ways: firstly, as suboptimal drop in BHCG level to 14% or less that necessitate second dose of methotrexate or surgical treatment; secondly, as the need for emergency laparoscopy or laparotomy in case of hemodynamic instability or severe abdominal pain. The following data were collected: demographic data including age, weight, and height, presenting symptoms, methotrexate dose given, ultrasound finding including ectopic size, fluid in pouch of Douglas, and BhCG level on day one and day 7 of methotrexate administration. BhCG level on the day of 1 at the time of methotrexate administration was called day 1. Some variation might be noted in the numbers of patients and that is due to some missing data. Bivariate associations were evaluated with use of odds ratio (OR) and Pearson's #2. Logistic regression was used to create an explanatory model for MTX failure. All analyses were performed with use of SPSS software (version 17).

## 3. Results

371 charts were reviewed. 146 subjects were excluded, 140 subjects were excluded because patients had surgery as primary mode of treatment, and 6 subjects were excluded because of the need for emergency surgery due to suspension of ruptured ectopic data after admission. 225 charts were included in the study and analyzed. The diagnosis of EP was based on the admitting physician assessment note. Dosing of methotrexate was based on body surface area (50 mg X BSA) in 151/225 (67%) of the patients; the rest received dose based on 1mg per kg body weight.

The average age was 30 years old. Before receiving methotrexate, most of the patients (68%) quantified their pain as moderate, only 29% were asymptomatic (Figure 3). Size of ectopic pregnancy on ultrasound was more than 4 cm in 58% of cases (Table 1).

**Figure 1:**
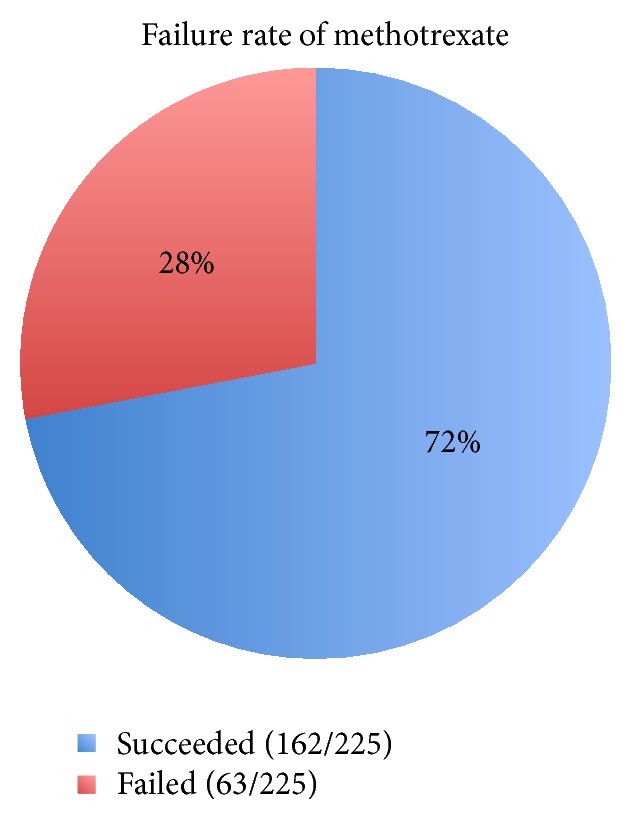
Failure rate of the 1st dose of methotrexate.

In 72% of the patients (162/225), single dose of methotrexate was successful; that is, BhCG decreased by more than 15% in the seventh day after administration. 28% (63/225) were labeled as failure of single dose of methotrexate because of suboptimal drop in BhCG (Figure 1).

**Figure 2:**
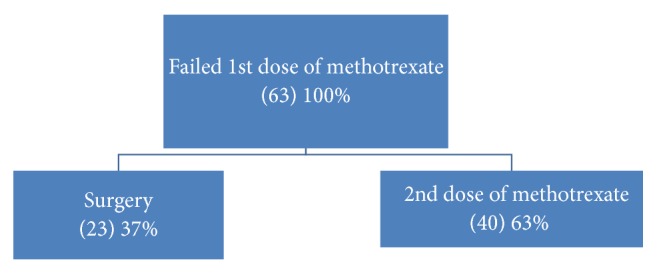
Modalities of treatment after failure methotrexate.

**Figure 3:**
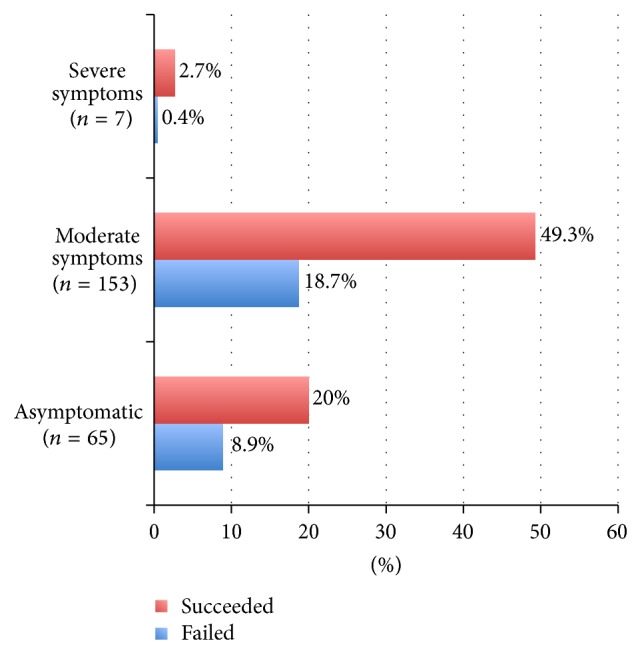
Symptomatology and failure of medical treatment.

63% (40/63) of failure received a second dose of methotrexate, and 37% (23/63) underwent surgical treatment (Figure 2).

The indication for surgical treatment were patient choice in 39%. Presence of pain 7 days after medical treatment 35% and rupture of ectopic 26% (Table 2).

**Figure 4:**
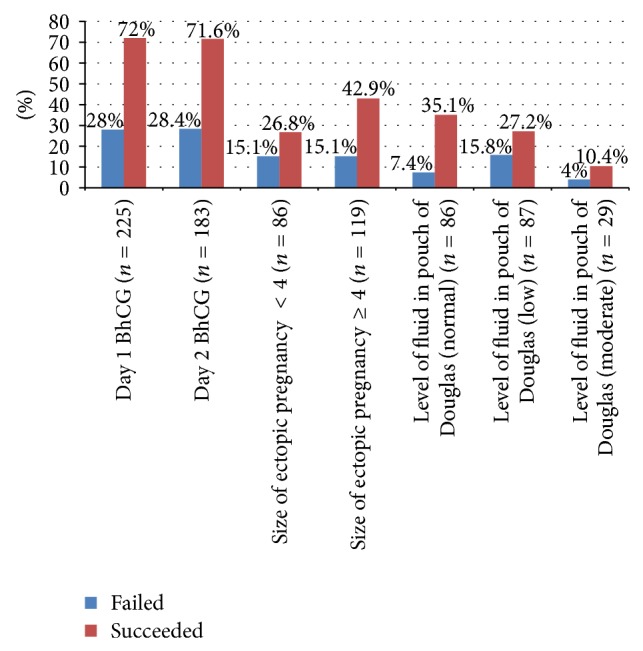
Predictors of failure of medical treatment.

Predictors for failure of single dose of methotrexate is Figure 4.

*(i) Initial Level of BhCG (Table 3)*. The higher the BHCG on day one, the lower is the failure rate (odds ratio of failure 0.3 95% CI (0.1–0.7); *P* value 0.004). In another term, with every increase log of beta HCG milli-international units per milliliter (mIU/mL) on day one, there is 60% chance of reducing likelihood of medical treatment failure.

*(ii) BHCG Day 7*. The higher is the levels of BhCG on day seven, the higher is the failure rate. (OR 3.66 95% CI 2.0–6.4; *P* value = 0.0001). With every increase log of beta HCG milli-international units per milliliter (mIU/mL) at day 7, there are 3.6 times higher odds of medical treatment failure. Mass size of ectopic pregnancy being ≥4 cm on U/S has not been shown to be statistically significant predictor of failure in our review.

## 4. Discussion

Methotrexate was first introduced as a successful treatment option for EP in the 1980's by Tanaka et al. [29]. Numerous published literatures have presented different success rates of medical management in resolving an EP; these rates were ranging from 85% to 95% [30, 31]. A study in Scandinavia showed a success rate of medical treatment in EP 76.2% [32]. Nguyen et al., 2010, described an outstandingly success rate of 100% (*n* = 30). In 2013, a similar cohort study presented a success rate of 89% [33]. Another study conducted in Makkah showed that all patients given methotrexate were successfully treated [34].

In this study the success rate of the medical management of ectopic pregnancy with methotrexate was found to be 72%. It appears to be relativity lower than international rates, an average of 90%; however our study had a remarkably higher success rate than a study conducted in Nigeria, which presented a success rate of only 3.8% [35]. This lower success rate in our study compared to the majority of equivalent studies may be attributed to the lack of a careful selection of patients as candidates for medical therapy and confining to a strict selection criterion. Among patient who received medical treatment, 71% had moderate or severe pain, and 58% had ectopic mass size of more than 4 cm.

As it has been established in a similar study conducted in Oman, following strict selection criteria raised the success rate of methotrexate therapy up to 20% more than their previous average success rate 40% [36].

Of those 28% who had failed medical treatment, 63% received a second dose of MTX, which is comparable to around 15–20% of women receiving a single dose of methotrexate who required a repeat dose of this chemotherapeutic agent in related studies [33–38], while the rest (37%) underwent surgical management either due to rupture of the EP, patient preference, or new onset of severe pain.

One of the risk factors of developing EP is being of age 40 years or older [27]. In this study, the mean age was 30 years, which is comparable to former studies [35–37]. Maternal age is not a predictor of MTX treatment in our study. It did however appear in a similar study that an increased maternal age will lead to a decrease in the in the success rate of MTX [36]. There were no mortalities in this study compared to the to a mortality report in the UK (issued in December 2007), showing a total of 10 deaths. These fatalities occurred during the nonsurgical management of an accurately established diagnosis of EP rather than because of an overlooked diagnosis or delayed surgical management, as these were the most common reasons for EP mortality during the previous four years [37, 38].

## Figures and Tables

**Table 1:** Baseline characteristics.

Variable	Mean (SD)

Age	30.3 (5.7)
Body weight (in kg)	72.7 (15.7)
Height (in centimeter)	154.2 (12.6)
Methotrexate dose (mg)	85.9 (12.7)

Clinical presentation	
Before starting treatment	
Asymptomatic	65 (28.9%)
Moderate pain	153 (68%)
Severe pain	7 (3.1%)

After receiving the 1st dose of methotrexate	
Asymptomatic	182 (80.8%)
Symptomatic	43 (19.1%)

Ectopic size on U/S	
Less than 4 cm	86 (42%)
More than 4 cm	119 (58%)

BHCG level	
Day 1	Mean 2219
Day 7	Mean 1802

**Table 2:** Indications for surgical treatment in patient whom failed first dose of methotrexate.

Indications	Number 23	Percentage
Patient refusal of second dose of MTX	9	39
Presence of pain on day 7 after medical treatment	8	35
Rupture of ectopic	6	26

**Table 3:** Association between level of BhCG and failure of medical treatment (multivariate regression analysis).

Variable	OR	95% OR CI	*P* value

BhCG day 1	0.3	0.1–0.7	0.004
Fluid in pouch of Douglas	0.4	0.1–1.1	0.1
Size of ectopic ≥4 cm	1.3	0.5–3.3	0.4
BhCG day 7	3.66	2.0–6.4	0.0001
